# Poverty and childhood malnutrition: Evidence-based on a nationally representative survey of Bangladesh

**DOI:** 10.1371/journal.pone.0256235

**Published:** 2021-08-23

**Authors:** Md. Ashfikur Rahman, Henry Ratul Halder, Md. Sazedur Rahman, Mahmood Parvez

**Affiliations:** 1 Development Studies Discipline, Social Science School, Khulna University, Khulna, Bangladesh; 2 Statistics Discipline, Science, Engineering and Technology School, Khulna University, Khulna, Bangladesh; 3 Rady Faculty of Health Sciences, Department of Community Health Sciences, University of Manitoba, Winnipeg, Manitoba, Canada; 4 BRAC James P Grant School of Public Health, BRAC University, Dhaka, Bangladesh; Yenepoya Medical College, Yenepoya University, INDIA

## Abstract

**Background:**

Malnutrition contributes to children’s morbidity and mortality, and the situation undermines the economic growth and development of Bangladesh. Malnutrition is associated with lower levels of education that decrease economic productivity and leads to poverty. The global burden of malnutrition continues to be unacceptably high amid social and economic growth, including in Bangladesh. Therefore, identifying the factors associated with childhood malnutrition and poverty is necessary to stop the vicious cycle of malnutrition leaded poverty.

**Methods:**

The study utilized the 2017–18 Bangladesh Demographic and Health Survey (BDHS), accumulating 7,738 mother-child pairs. Associations between potential risk factors and nutritional status were determined using chi-square tests, and multivariate logistic regression models were utilized on significant risk factors to measure their odds ratio (OR) with their 95% confidence intervals (CI).

**Results:**

The prevalence of moderate and severe wasting was 7.0% and 1.8%, respectively, whereas the prevalence of moderate and severe stunting was 19.2% and 8.0%, while 16.4% and 3.6% of children were moderately and severely underweight. Children from the poorest and poor households were suffering from at least one form of malnutrition. Adjusted ORs were estimated by controlling socio-economic and demographic risk factors, such as poor maternal body mass index, parents’ lower education level, use of unhygienic toilet, child age in months, and recent experience of diarrhea and fever. The pattern was almost similar for each malnutrition status (i.e., stunting, underweight, and wasting) in the poorest and poor households.

**Conclusion:**

Bangladesh achieved the Millennium Development Goals, focusing primarily on health-related indicators and working to achieve the Sustainable Development Goals. Even considering this success, the prevalence of malnutrition and poverty in same household remains relatively high compared to other developing countries. Therefore, the study recommends the implementation of nationwide systematic measures to prevent poverty and malnutrition.

## Background

Worldwide, childhood malnutrition is a significant public health concern as it contributes to impaired mental and physical growth and is a significant cause of child morbidity and mortality [1–3]. Around 3.5 million children die every year from malnutrition, and low- and middle-income countries (LMICs) are more prevalent, advancing the global burden of diseases by 11% [4]. Furthermore, the 2021 joint report of childhood malnutrition by the United Nations Children’s Fund, World Health Organization (WHO), and World Bank Group found that 149 and 45.4 million children under age five in 2020 were stunted and wasted [5].

Poverty and childhood malnutrition are believed to be interlinked [6], which is mediated by inadequate diet plan, lower education level, poor living standards, and lack/no access to health facilities, safe water, proper sanitation and hygiene [6, 7]. Hence, developing countries, including Bangladesh, prioritize reducing poverty and childhood malnutrition by various policy implications. Besides, a poorly nourished child has a greater likelihood of being less productive in his/her adulthood, adversely impacting the economy in the long run [8]. Therefore, proper nutrition in childhood is the foremost need as it increases their survival probability and ensures a better economy of a country.

As the effects of childhood malnutrition are intergenerational, public policies and poverty reduction strategies have targeted multi-faceted approaches in developing countries [7, 9]. Bangladesh has already initiated several of these approaches despite accounting for their effectiveness, such as nutritional programs (i.e., food for education and nutrition), education for poverty reduction [1, 2, 7, 9]. Moreover, the Poverty Reduction Strategy Paper (PRSP) of Bangladesh designed various plans to achieve strategic and employment growth, implementing macroeconomic structure in public and private sectors. The PRSP plan that includes strategic human resources development aims to reach the poor and vulnerable population for the protection of the environment, climate change, and disaster management [10]. Over the past fifteen years, Bangladesh has succeeded in reducing childhood malnutrition, but substantial inequalities exist across geographical regions and economic groups [8, 11]. According to 2014 [12] and 2017–18 [13] Bangladesh Demographic and Health Survey (BDHS), children from the lowest quintile were most prevalent in stunting, wasting, and underweight; whereas, the scenario is the total opposite for the highest quintile.

Many studies have documented the relationship between childhood malnutrition and wealth quintiles-based measures utilizing Demographic Health Surveys (DHS) data, demonstrating that childhood malnutrition is still higher in the poor quintile than the non-poor counterparts [7, 14–16]. However, apart from economic factors, many non-economic factors (such as place of residence, geographical locations, access to water and sanitation, maternal and child-related biological characteristics, etc.) are also regarded as important determinants of childhood malnutrition at the household level [1–3, 7, 17, 18]. To promote health equity and equitable gains, policymakers and development professionals strongly emphasize reducing this economic gap to have a more significant impact on childhood malnutrition [19].

In light of the discussion, it is essential for the government and development partners to carefully examine and identify the significant determinants of childhood malnutrition and its linkage to poverty [19]. However, only a few studies have focused on the determinants linked to this issue. This research will allow the government and development practitioners to curate strategic decisions, reduce the prevalence of childhood malnutrition, and design and follow-up effective actions for both conditions.

## Methods

### Data sources

**Fig 1:**
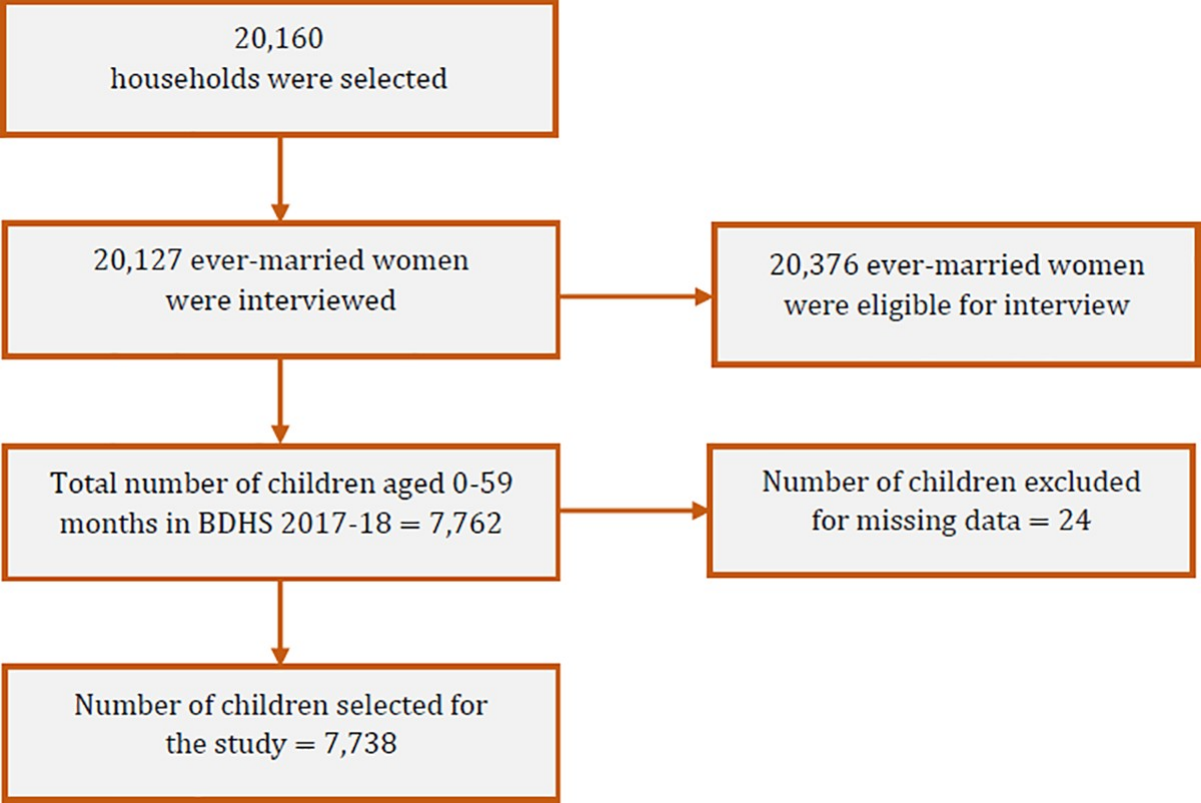
Sampling flowchart for the selection of participants.

The study used the 2017–18 BDHS data, which is freely available upon request. The survey was conducted between October 2017 and March 2018 under the National Institute of Population Research and Training, Medical Education and Family Welfare Division, and Ministry of Health and Family Welfare to assess health indicators and provide a detailed overview of the Bangladeshi population, and maternal and child health-related issues. A total of 7,738 mother-child pairs’ information was assessed in the current study. Detailed survey information (such as data collection procedures, sample size determination, etc.) was described in the BDHS 2017–18 report [13]. The study included ever-married women aged 15–49 with valid body mass index (BMI) who are currently not pregnant and gave birth to at least one child preceding the survey. Unmarried and not pregnant mothers with incomplete BMI information were excluded from the sample. A total of 20,127 ever-married women aged between 15 and 49 years were interviewed out of 20,376 eligible women [13]. Only 7,762 participants gave birth to children, and there were 24 missing values among them. We excluded the missing values from our analyses, making the total number of observations 7,738 (Fig 1).

### Study variables

#### Dependent variables

We used three dependent variables for this study: stunting, underweight, and wasting. Dependent variables were classified based on Z-scores of height-for-ages (HAZ) for stunting, weight-for-height (WHZ) for wasting, and weight-for-age (WAZ) for underweight. WHO Anthro Plus (version 3.2.2) was used to calculate the Z-scores and they were categorized either as nourished (Z-score ≥ −2.0), moderately stunted/underweight/wasted (-3.0 ≤ Z-score < -2.0), or severely stunted/underweight/wasted (Z-score < -3.0) following WHO Child Growth Standards guideline [20]. In this study, a small number of children were severely malnourished; therefore, moderate and severe malnutrition were merged into one. The outcome variables used in this study are stunting, underweight, and wasting (coded 1 = presence of the condition, otherwise 0).

#### Independent variables

The study analyzed independent variables from prior literature, which included three main categories: socioeconomic and demographic characteristics, child-related determinants, and paternal characteristics. Socioeconomic and demographic characteristics involved: division (Barisal, Chittagong, Dhaka, Khulna, Mymensingh, Rajshahi, Rangpur, Sylhet); place of residence (urban, rural); drinking water source (unimproved, improved); type of toilet (unhygienic, hygienic); place of childbirth (facility birth, home delivery) [21, 22]. Child-related determinants included birth order (1^st^, 2^nd^, 3^rd^, ≥4^th^); age in months (0–5, 6–11, 12–23, 24–35, 36–59); sex (male, female); experience of diarrhea and fever two weeks preceding the survey (no, yes). Furthermore, paternal factors consist of maternal BMI (underweight: <18.50kg/m^2^, normal: 18.50–24.99kg/m^2^, overweight/obese: ≥25.00kg/m^2^) based on the global cut-off points [23]; maternal age in years (15–24, 25–34, 35–49); maternal age at first birth (<18, 19–24, ≥25); parental education (no education, primary, secondary, higher); mother’s current working status (no, yes); mother’s exposure to television (no, yes); mother’s antenatal care (ANC) visit number (Nil, 1–3, ≥4); father’s occupation (agricultural, business, non-agricultural, other). Household wealth status was originally categorized into five groups based on principal component analysis, which was then recoded into three groups: poorest, poor, and well-off (consisted of medium, richest, and richer).

### Statistical analyses

Before conducting any statistical analyses, we weighted the dataset due to the cluster sampling design of DHS. Then, we reported descriptive statistics (i.e., frequency and percentage) and weighted prevalence with their 95% confidence intervals (CIs) of selected independent variables. Pearson’s chi-square test of independence examined the relationship between dependent and independent variables. We implemented multivariate logistic regression models to estimate the adjusted odds ratios (AORs) and their 95% CIs for stunting, underweight, and wasting segregated by wealth status (i.e., poorest, poor, and well-off). Besides, all confounding variables were controlled while multivariate modeling, and we examined multicollinearity among independent variables by applying the variance inflation factor. All data analyses were performed using the Statistical Package for Social Science (SPSS) version 25 (SPSS Inc., Chicago, USA) and R version 4.0.2 (Bell Laboratories, New Jersey, USA).

#### Ethical consideration

DHS data exists in the public domain and is freely accessible upon reasonable request. The Ethics Committee of Bangladesh and ICF international approved the study protocol; therefore, we did not require further ethical approval. Details of the ethical approval can be found in the BDHS 2017–18 summary report [13].

## Results

**Table 1** exhibits the univariate results of socioeconomic and demographic, parental, and child-related characteristics from 7,738 children aged under five years. 7% and 1.8% of children were moderately and severely wasted, whereas moderate and severe stunting percentages were 19.2% and 8.0%, respectively. Results also showed, 16.4% and 3.6% of children were moderately and severely underweight. More than half (52.3%) of the children were male, the majority (73.6%) were from rural areas, almost one-fourth (24.9%) were from Dhaka, and about 7.0% of mothers and 15.2% of fathers had no formal education.

**Table 1:** Frequency distribution of background characteristics for study participants (n = 7,738).

Background Characteristics		Total n (%)	Poorest n (%)	Poor n (%)	Well-Off n (%)
**Child-related Characteristics**					
**Malnutrition**					
	Moderate Wasting	538 (7.0)	139 (8.2)	111 (7.0)	288(6.4)
	Severe Wasting	142 (1.8)	33 (2.0)	27 (1.7)	82(1.8)
	Moderate Stunting	1484 (19.2)	408 (24.2)	387 (15.4)	689(15.4)
	Severe Stunting	619 (8.0)	207 (12.3)	140 (8.9)	277(6.1)
	Moderate Underweight	1265 (16.4)	364 (21.6)	290 (18.3)	611(13.7)
	Severe Underweight	276 (3.6)	91 (5.4)	70 (4.4)	115(2.6)
**Birth Order**					
	1^st^	2930 (37.9)	493 (29.2)	547 (34.6)	1890(42.3)
	2^nd^	2535 (32.8)	517 (30.6)	522 (33.0)	2535(32.8)
	3^rd^	1310 (16.9)	327 (19.4)	286 (18.1)	1310(16.9)
	≥4^th^	961 (12.4)	350 (20.7)	226 (14.3)	961(12.4)
**Age (months)**					
	0–5	913 (11.8)	193 (11.4)	178 (11.3)	542(12.1)
	6–11	789 (10.2)	169 (10.0)	185 (11.7)	435(9.7)
	12–23	1612 (20.8)	331 (19.6)	342 (21.6)	939(21.0)
	24–35	1507 (19.5)	328 (19.4)	301 (19.0)	878(19.7)
	36–59	2916 (37.7)	668 (39.6)	575 (36.4)	1673(37.5)
**Sex**					
	Male	4047 (52.3)	885 (52.5)	826 (52.3)	2336(52.3)
	Female	3688 (47.7)	802 (47.5)	754 (47.7)	2132(47.7)
**Recent Experience of Diarrhea** ^ϯ^					
	No	7360 (95.2)	1606 (95.2)	1505 (95.3)	4249(95.1)
	Yes	375 (4.8)	81 (4.8)	75 (4.7)	219(4.9)
**Recent Experience of Fever** ^ϯ^					
	No	5129 (66.3)	1099 (65.1)	1047 (66.3)	2983(66.8)
	Yes	2606 (33.7)	588 (34.9)	533 (33.7)	1485(33.2)
**Socio-economic and Demographic Characteristics**					
**Division**					
	Barisal	435 (5.6)	156 (9.2)	105 (6.6)	174(3.9)
	Chittagong	1601 (20.7)	296 (17.5)	276 (17.5)	1029(23.0)
	Dhaka	1925 (24.9)	203 (12.0)	243 (15.4)	1479(33.1)
	Khulna	724 (9.4)	107 (6.3)	161 (10.2)	456(10.2)
	Mymensingh	660 (8.5)	207 (12.3)	189 (12.0)	264(5.9)
	Rajshahi	901 (11.6)	193 (11.4)	230 (14.6)	478(10.7)
	Rangpur	842 (10.9)	334 (19.8)	225 (14.2)	283(6.3)
	Sylhet	648 (8.4)	192 (11.4)	150 (9.5)	306(6.8)
**Place of Residence**					
	Urban	2040 (26.4)	185 (11.0)	133 (8.4)	1722(38.5)
	Rural	5695 (73.6)	1502 (89.0)	1447 (91.6)	2746(61.5)
**Drinking Water Source**					
	Unimproved	83 (1.1)	24 (1.4)	31 (2.0)	28(0.6)
	Improve	7654 (98.9)	1664 (98.6)	1550 (98.0)	4440(99.4)
**Type of Toilet**					
	Unhygienic	2165 (28.0)	1018 (60.3)	679 (43.0)	468(10.5)
	Hygienic	5571 (72.0)	670 (39.7)	901 (57.0)	4000(89.5)
**Place of Childbirth**					
	Facility Birth	2398 (49.7)	270 (26.5)	373 (37.1)	1755(62.7)
	Home Delivery	2425 (50.3)	750 (73.5)	633 (62.9)	1042(37.3)
**Parental Characteristics**					
**Maternal BMI**					
	Underweight (<18.50kg/m^2^)	1074 (13.9)	356 (21.1)	283 (17.9)	435(9.7)
	Normal (18.50–24.99kg/m^2^)	4680(60.5)	1128(66.8)	1057(66.9)	2495(55.8)
	Overweight/Obese (≥25.00kg/m^2^)	1983 (25.6)	204 (12.1)	241 (15.2)	1538(34.4)
**Maternal Age (years)**					
	15–24	3687 (47.7)	784 (46.5)	812 (51.4)	2091(46.8)
	25–34	3472 (44.9)	767 (45.5)	652 (41.2)	2053(45.9)
	35–49	577 (7.5)	136 (8.1)	117 (7.4)	324(7.3)
**Maternal Age at First Birth (years)**					
	<18	4532 (58.6)	1194 (70.7)	1060 (67.0)	2278(51.0)
	19–24	2844 (36.8)	466 (27.6)	488 (30.9)	1890(42.3)
	≥25	361 (4.7)	28 (1.7)	33 (2.1)	300(6.7)
**Mother’s Education**					
	No Education	542 (7.0)	243 (14.4)	117 (7.4)	182(4.1)
	Primary	2214 (28.6)	795 (47.1)	563 (35.6)	856(19.2)
	Secondary	3790 (49.0)	604 (35.8)	810 (51.2)	2376(53.2)
	Higher	1192 (15.4)	46 (2.7)	91 (5.8)	1055(23.6)
**Mother’s Current Working Status**					
	No	4640 (60.0)	779 (46.2)	801 (50.7)	3060(68.5)
	Yes	3098 (40.0)	908 (53.8)	780 (49.3)	1408(31.5)
**Mother’s Exposure to Television**					
	No	2868 (37.1)	1268 (75.2)	750 (47.4)	850(19.0)
	Yes	4868 (62.9)	419 (24.8)	831 (52.6)	3618(81.0)
**Mother’s ANC Visit Number**					
	Nil	3478 (45.0)	881 (52.2)	722 (45.7)	1875(42.0)
	1–3	2061 (26.6)	505 (29.9)	503 (31.8)	1053(23.6)
	≥4	2197 (28.4)	302 (17.9)	355 (22.5)	1540(34.5)
**Father’s Education**					
	No Education	1176 (15.2)	524 (31.1)	297 (18.8)	355(7.9)
	Primary	2677 (34.6)	792 (46.9)	729 (46.1)	1156(25.9)
	Secondary	2556(33.0)	314(18.6)	457(28.9)	1785(40.0)
	Higher	1327 (17.20)	57 (3.4)	98 (6.2)	1172(26.2)
**Father’s Occupation**					
	Agricultural	1589 (20.5)	626 (37.1)	445 (28.1)	518(11.6)
	Business	1649 (21.3)	203 (12.0)	268 (17.0)	1178(26.4)
	Non-Agricultural	3533 (45.7)	523 (31.0)	636 (40.2)	2374(53.1)
	Other	967 (12.5)	336 (19.9)	232 (14.7)	399(8.9)

^ϯ^ Recent experience indicates sufferings from diarrhea and fever two weeks preceding the survey.

BMI: Body mass index; ANC: Antenatal care.

**Table 1** also portrays the frequency and percentage of selected background characteristics for under-five children segregated by household wealth status. All forms of child malnutrition (i.e., wasting, stunting, and underweight) peaked in the poorest households and the lowest in well-off families. Poorest households had more frequency of underweight mothers, whereas overweight/obese mothers were more frequent in well-off families. More than 70% of mothers who gave their first birth before 18 years were from the poorest families, while the percentage was much lower in poor (67.0%) and well-off (51.0%) households. Percentages of parents with secondary and higher education, access to hygienic toilets, and facility birth were considerably higher in well-off families compared to their poorest and poor counterparts. Mothers’ ANC visit number also increased with better household status.

**Table 2** shows the prevalence of wasting, stunting, and underweight by the selected characteristics of under-five children with their 95% CIs. Results show that the prevalence of all forms of malnutrition (i.e., wasting, stunting, and underweight) was the highest in Sylhet division, poorest households, and underweight, illiterate mothers aged between 35 and 49 years. Mothers’ first birth age of <18 years, lack of exposure to television, households with unimproved water, and unhygienic toilet facilities also had a higher prevalence for all forms of malnutrition. The prevalence of wasting, stunting, and underweight was the highest amongst male children, children born at home, and who had recent experience of fever. Moreover, the prevalence of wasting was the highest among children aged below six months. While 24–35 and 36–59 months aged children were more prevalent in stunting and underweight, receptively.

**Table 2:** Prevalence of malnutrition by background characteristics with 95% confidence interval (n = 7,738).

Independent Variables		Wasting (95% CI)	Stunting (95% CI)	Underweight (95% CI)
**Child-related Variables**				
**Birth Order**				
	1^st^	8.7 (7.5–10.1)	25.0 (23.1–26.9)	17.9 (15.3–19.6)
	2^nd^	8.9 (7.7–10.3)	25.0 (22.9–27.3)	18.8 (17.1–20.7)
	3^rd^	8.9 (7.4–10.7)	28.8 (26.0–31.7)	20.5 (18.2–23.0)
	≥4^th^	8.3 (6.6–10.4)	37.4 (33.9–41.1)	28.2 (24.8–31.8)
**Age (months)**				
	0–5	10.2 (8.1–12.9)	10.5 (8.4–13.1)	9.2 (7.4–11.5)
	6–11	6.9 (5.2–9.0)	15.4 (12.8–18.5)	12.5 (10.1–15.3)
	12–23	8.4 (7.0–10.2)	30.3 (27.7–33.1)	16.9 (15.0–19.1)
	24–35	8.8 (7.3–10.7)	36.1 (33.0–39.3)	23.3 (20.8–26.0)
	36–59	9.0 (7.9–10.3)	29.2 (27.1–31.4)	25.2 (23.2–27.2)
**Sex**				
	Male	9.6 (8.6–10.8)	27.4 (25.7–29.1)	20.0 (18.4–21.7)
	Female	7.9 (6.9–9.0)	27.0 (25.1–28.9)	19.8 (18.3–21.4)
**Recent Experience of Diarrhea** ^ϯ^				
	No	8.7 (8.0–9.6)	27.3 (25.9–28.7)	19.9 (18.7–21.1)
	Yes	9.6 (6.8–13.4)	25.3 (20.3–30.9)	20.9 (16.8–25.6)
**Recent Experience of Fever** ^ϯ^				
	No	7.8 (7.0–8.8)	27.2 (25.6–28.8)	18.5 (17.2–19.9)
	Yes	10.6 (9.3–12.2)	27.2 (24.9–29.6)	22.7 (20.7–24.8)
**Socioeconomic and Demographic Variables**				
**Division**				
	Barisal	9.1 (7.0–11.7)	29.1 (25.3–33.2)	19.9 (16.7–23.4)
	Chittagong	8.1 (6.4–10.2)	29.6 (26.0–33.5)	19.6 (16.9–22.5)
	Dhaka	9.5 (7.6–11.9)	22.6 (19.3–26.1)	17.6 (14.7–20.9)
	Khulna	8.1 (6.4–10.1)	22.5 (19.2–26.2)	17.4 (14.7–20.4)
	Mymensingh	9.7 (7.9–11.9)	30.7 (27.1–34.6)	23.7 (20.9–26.6)
	Rajshahi	8.3 (6.5–10.7)	26.9 (23.6–30.4)	20.4 (16.8–24.5)
	Rangpur	7.3 (5.7–9.2)	25.7 (22.9–28.9)	17.6 (15.1–20.5)
	Sylhet	10.5 (8.9–12.3)	37.6 (33.9–41.3)	29.0 (25.9–32.3)
**Place of Residence**				
	Urban	9.7 (8.2–11.4)	21.7 (19.3–24.2)	18.4 (16.3–20.6)
	Rural	8.5 (7.6–9.4)	29.2 (27.5–30.9)	20.5 (19.1–22.0)
**Household Wealth Status**				
	Poorest	10.2 (8.8–11.9)	36.4 (33.9–39.0)	27.0 (24.5–29.6)
	Poor	8.7 (7.3–10.4)	33.3 (30.5–36.2)	22.8 (20.4–25.3)
	Well-Off	8.3 (7.2–9.4)	21.5 (19.8–23.3)	16.2 (14.9–17.7)
**Drinking Water Source**				
	Unimproved	11.5 (7.4–17.5)	40.0 (29.1–51.9)	27.9 (18.9–39.2)
	Improved	8.8 (8.0–9.6)	27.0 (25.6–28.5)	19.8 (18.6–21.1)
**Type of Toilet**				
	Unhygienic	9.5 (8.3–11.0)	34.1 (31.9–36.4)	25.6 (23.2–29.2)
	Hygienic	8.5 (7.6–9.5)	24.5 (22.9–26.2)	17.7 (16.5–19.0)
**Place of Childbirth**				
	Facility Birth	8.1 (6.8–9.7)	20.7 (18.9–22.6)	13.1 (11.6–14.7)
	Home Delivery	9.1 (7.9–10.6)	31.2 (29.0–33.4)	20.4 (18.5–22.3)
**Parental Variables**				
**Maternal BMI**				
	Underweight (<18.50kg/m^2^)	14.1 (12.1–16.5)	37.2 (34.1–40.3)	29.0 (26.1–32.1)
	Normal (18.50–24.99kg/m^2^)	8.4 (7.4–9.4)	27.6 (25.9–29.4)	20.0 (18.5–21.5)
	Overweight/Obese (≥25.00kg/m^2^)	6.9 (5.6–8.4)	20.7 (18.6–23.1)	14.9 (13.0–16.9)
**Maternal Age (years)**				
	15–24	8.7 (7.7–9.9)	26.6 (24.7–28.5)	18.4 (16.9–20.1)
	25–34	8.7 (7.7–9.9)	27.7 (25.8–29.7)	20.9 (19.2–22.6)
	35–49	9.5 (7.2–12.4)	27.9 (24.3–31.8)	23.7 (19.9–27.9)
**Maternal Age at First Birth (years)**				
	<18	9.3 (8.3–10.3)	29.7 (27.9–31.4)	21.9 (20.3–23.5)
	19–24	8.3–7.1–9.6)	24.7 (22.7–26.8)	17.6 (16.0–19.3)
	≥25	7.0 (4.5–10.8)	15.8 (12.1–20.3)	13.6 (10.1–17.9)
**Mother’s Education**				
	No Education	12.3 (9.7–15.5)	40.4 (35.7–45.3)	34.5 (29.7–39.6)
	Primary	9.5 (8.2–10.9)	34.6 (32.2–37.1)	24.4 (22.1–26.7)
	Secondary	8.7 (7.6–9.9)	25.7 (23.8–27.7)	18.5 (17.1–20.1)
	Higher	6.2 (4.8–8.1)	12.1 (10.2–14.4)	9.5 (7.7–11.5)
**Mother’s Current Working Status**				
	No	8.8 (7.8–9.9)	24.7 (23.0–26.5)	18.5 (17.1–20.0)
	Yes	8.8 (7.8–9.8)	30.9 (29.0–33.0)	22.0 (20.3–23.9)
**Mother’s Exposure to Television**				
	No	9.0 (7.9–10.2)	33.0 (30.9–35.1)	23.7 (21.8–25.7)
	Yes	8.7 (7.7–9.7)	23.8 (22.2–25.4)	17.7 (16.4–19.1)
**Mother’s ANC visit Number**				
	Nil	8.7 (7.7–9.9)	30.2 (28.3–32.2)	24.9 (23.1–26.8)
	1–3	9.2 (7.8–10.8)	27.7 (25.4–30.1)	17.3 (15.4–19.3)
	≥4	8.5 (7.2–10.0)	21.9 (19.9–24.0)	14.5 (12.9–16.3)
**Father’s Education**				
	No Education	9.7 (8.1–11.6)	40.1 (37.0–43.3)	28.9 (25.8–32.2)
	Primary	8.9 (7.8–10.1)	31.6 (29.6–33.7)	22.4 (20.6–24.3)
	Secondary	9.1 (7.8–10.6)	23.7 (21.7–25.8)	17.7 (16.0–19.6)
	Higher	7.2 (5.7–9.0)	13.6 (11.5–16.0)	11.1 (9.3–13.3)
**Father’s Occupation**				
	Agricultural	9.8 (8.4–11.5)	34.4 (31.7–37.1)	24.3 (21.7–27.1)
	Business	6.8 (5.5–8.2)	23.0 (20.5–25.7)	16.9 (14.9–19.0)
	Non-Agricultural	8.9 (7.8–10.2)	23.5 (21.8–25.3)	17.8 (16.3–19.4)
	Other	10.1 (8.1–12.5)	35.9 (32.3–39.5)	25.7 (22.7–28.9)

^ϯ^ Recent experience indicates sufferings from diarrhea and fever two weeks preceding the survey.

CI: Confidence interval; BMI: Body mass index; ANC: Antenatal care.

**Table 3** represents associated factors of stunting segregated by household wealth status. The result indicated that underweight mothers significantly affected the poorest and poor households due to higher ORs. Poorer households showed a significant association of stunting with division, indicating a greater likelihood of stunted children in Chittagong (1.16 folds higher than Sylhet). However, maternal age at first birth was not significantly associated with stunting among various household wealth statuses. Paternal education greatly impacted poor and rich households (2.94 and 1.98 times more than higher education levels), which resulted in stunted children. The odds of stunting were higher in rich households for fathers with non-agricultural services and business. In poorer households, 1^st^, 2^nd^, and 3^rd^ birth order children had a lower odds of being stunted when compared to ≥4^th^ birth order. Children’s age significantly affected all households; for example, the likelihood of stunting was higher for children aged 36 to 59 months in the poorest and poor families, but odds were lower in well-off families. Low maternal BMI was significantly associated with increased stunting for the poorest and poor compared with well-off families.

**Table 3:** Factors associated with stunting within different wealth status.

		Poorest		Poor		Well-off	
Independent Variables		AOR (95% CI)	p-value	AOR (95% CI)	p-value	AOR (95% CI)	p-value
**Child-related Variables**							
**Birth Order (Ref: ≥4** ^**th**^ **)**							
	1^st^	0.93(0.68–1.29)	0.677	0.65(0.39–1.07)	0.090	0.91(0.63–1.32)	0.615
	2^nd^	0.87(0.64–1.19)	0.382	0.58(0.38–0.89)	0.012	0.86(0.60–1.23)	0.405
	3^rd^	0.92(0.66–1.29)	0.639	0.68(0.45–1.03)	0.067	0.93(0.63–1.37)	0.700
**Age (months) (Ref: 0–5)**							
	6–11	1.08(0.63–1.86)	0.782	0.11(0.05–0.22)	<0.001	0.10(0.01–0.81)	0.031
	12–23	2.92(0.63–1.86)	<0.001	0.38(0.22–0.67)	0.001	0.14(0.02–1.11)	0.062
	24–35	4.41(1.88–4.55)	<0.001	1.23(0.71–1.79)	0.616	0.34(0.04–2.63)	0.300
	36–59	3.11(1.90–5.09)	<0.001	1.62(1.06–2.47)	0.026	0.37(0.5–2.91)	0.348
**Socioeconomic and Demographic Variables**							
**Division (Ref: Sylhet)**							
	Barisal	0.83(0.53–1.30)	0.413	N/A	N/A	1.10(0.72–1.69)	0.652
	Chittagong	1.16(0.79–1.71)	0.445	N/A	N/A	1.04(0.74–1.45)	0.819
	Dhaka	0.77(0.51–1.18)	0.229	N/A	N/A	0.79(0.55–1.13)	0.191
	Khulna	0.41(0.26–0.71)	0.001	N/A	N/A	1.01(0.67–1.50)	0.981
	Mymensingh	0.75(0.50–1.14)	0.184	N/A	N/A	1.01(0.66–1.52)	0.997
	Rajshahi	0.58(0.37–0.89)	0.014	N/A	N/A	1.02(0.68–1.54)	0.916
	Rangpur	0.52(0.35–0.77)	0.001	N/A	N/A	1.11(0.71–1.75)	0.645
**Place of residence (Ref: Rural)**							
	Urban	N/A	N/A	N/A	N/A	0.94(0.76–1.16)	0.543
**Drinking Water Source (Ref: Improved)**							
	Unimproved	N/A	N/A	N/A	N/A	1.33(0.54–3.29)	0.540
**Type of Toilet (Ref: Hygienic)**							
	Unhygienic	N/A	N/A	1.06(0.84–1.33)	0.624	1.12(0.83–1.50)	0.474
**Place of Childbirth (Ref: Home Delivery)**							
	Facility Birth	N/A	N/A	N/A	N/A	0.89(0.72–1.11)	0.307
**Parental Variables**							
**Maternal BMI (Ref: Overweight/Obese)**							
	Underweight (<18.50kg/m^2^)	1.6(1.09–2.35)	0.016	2.11(1.43–2.23)	<0.001	1.35(0.96–1.90)	0.083
	Normal (18.50–24.99kg/m^2^)	1.14(0.81–1.59)	0.458	1.70(1.21–2.39)	0.002	1.02(0.81–1.29)	0.850
**Maternal Age at First Birth (Ref: <18 years)**							
	19–24	N/A	N/A	N/A	N/A	0.90(0.73–1.11)	0.332
	≥25	N/A	N/A	N/A	N/A	0.91(0.58–1.42)	0.662
**Mother’s Education (Ref: Higher)**							
	No Education	1.99(0.83–4.80)	0.123	2.41(1.22–6.58)	0.016	1.70(0.96–3.02)	0.071
	Primary	1.60(0.70–3.66)	0.269	3.72(1.78–7.67)	<0.001	1.55(1.05–2.72)	0.027
	Secondary	1.66(0.73–3.78)	0.226	3.51(1.70–6.94)	0.001	1.41(1.03–1.91)	0.030
**Mother’s Current Working Status (Ref: Yes)**							
	No	N/A	N/A	N/A	N/A	0.88(0.70–1.90)	0.241
**Mother’s Exposure to Television (Ref: No)**							
	Yes	N/A	N/A	N/A	N/A	0.99(0.78–1.27)	0.985
**Mother’s ANC Visit Number (Ref: ≥4)**							
	Nil	0.76(0.51–1.14)	0.296	1.16(0.74–1.84)	0.514	1.22(0.84–1.77)	0.295
	1–3	0.70(0.51–0.98)	0.028	1.23(0.89–1.72)	0.214	1.20(0.97–1.49)	0.093
**Father’s Education Level (Ref: Higher)**							
	No Education	2.94(1.37–6.34)	0.006	1.45(0.81–2.60)	0.216	1.98(1.26–3.11)	0.003
	Primary	2.41(1.15–5.10)	0.021	1.01(0.59–1.74)	0.960	1.89(1.34–2.67)	<0.001
	Secondary	2.18(1.02–4.67)	0.045	0.87(0.50–1.50)	0.614	1.51(0.12–2.04)	0.008
**Father’s Occupation (Ref: Other)**							
	Agricultural	N/A	N/A	N/A	N/A	0.82(0.54–1.25)	0.351
	Business	N/A	N/A	N/A	N/A	0.70(0.48–1.01)	0.055
	Non-Agricultural	N/A	N/A	N/A	N/A	0.71(0.51–1.00)	0.050

N/A stands for not applicable. Insignificant variables in Chi-square tests were not included in the adjusted model; therefore, they were replaced with N/A in the Table.

p-value <0.05 is the level of significance.

AOR: Adjusted odds ratio; p-value: Probability value; CI: Confidence interval; Ref: Reference category; BMI: Body mass index; ANC: Antenatal care.

**Table 4** visualizes children’s odds of being underweight by household wealth statuses (i.e., poor, poorer, and well-off). Results implied that maternal BMI significantly affected all wealth statuses. For instance, if a mother was underweight, there was a higher likelihood of the child being underweight. Besides, illiterate parents from the poorest and poor households had higher chances to have an underweight child. In poorest households of Dhaka and Barisal, there was a greater chance for a child being underweight, but the likelihood was higher for poor households in Mymensingh. Furthermore, maternal age, mother’s exposure to television, drinking water source, type of toilet, mother’s ANC visit number, child’s birth order, place of childbirth showed insignificant association with underweight within different wealth statuses.

**Table 4:** Factors associated with underweight within different categories of wealth status.

		Poorest		Poor		Well-Off	
Independent Variables		AOR (95% CI)	p-value	AOR (95% CI)	p-value	AOR (95% CI)	p-value
**Child-related Variables**							
**Birth Order (Ref: ≥4)**							
	1^st^	1.55(0.83–2.90)	0.864	N/A	N/A	0.88(0.51–1.52)	0.634
	2^nd^	1.04(0.60–1.80)	0.621	N/A	N/A	0.74(0.47–1.17)	0.197
	3^rd^	0.97(0.57–1.63)	0.552	N/A	N/A	0.71(0.45–1.12)	0.137
**Age (Ref: 0–5 months)**							
	6–11	1.68(0.90–3.16)	<0.001	2.60(1.27–5.34)	0.001	0.24(0.02–2.51)	0.231
	12–23	2.95(1.71–5.08)	0.050	3.11(1.61–6.00)	0.001	0.21(0.02–2.30)	0.204
	24–35	3.50(2.05–6.00)	0.811	5.72(0.98–6.01)	0.193	0.29(0.03–3.05)	0.302
	36–59	1.68(0.90–3.16)	0.290	7.37(3.49–15.55)	0.001	0.51(0.05–5.29)	0.568
**Recent Experience of Fever (Ref: No)**							
	Yes	N/A	N/A	1.51(1.16–1.95)	0.002	1.30(1.03–1.64)	0.027
**Socioeconomic and Demographic Variables**							
**Division (Ref: Sylhet)**							
	Barisal	1.07(0.56–2.03)	0.995	0.53(0.28–0.99)	0.040	0.55(0.32–0.94)	0.030
	Chittagong	0.91(0.52–1.59)	0.771	0.55(0.34–0.89)	0.008	0.89(0.61–1.30)	0.538
	Dhaka	1.06(0.56–2.02)	0.523	0.62(0.38–1.01)	0.036	0.68(0.46–1.01)	0.058
	Khulna	0.82(0.37–1.82)	0.242	0.67(0.39–1.15)	0.122	0.78(0.50–1.22)	0.278
	Mymensingh	0.85(0.46–1.57)	0.864	0.85(0.52–1.40)	0.367	0.84(0.53–1.32)	0.444
	Rajshahi	0.60(0.30–1.21)	0.412	0.58(0.35–0.96)	0.037	0.74(0.46–1.17)	0.196
	Rangpur	0.79(0.45–1.41)	0.044	0.42(0.25–0.71)	0.001	0.86(0.52–1.43)	0.554
**Place of Residence (Ref: Urban)**							
	Rural	N/A	N/A	0.71(0.47–1.08)	0.079	N/A	N/A
**Type of Toilet (Ref: Hygienic)**							
	Unhygienic	1.25(0.90–1.74)	0.301	N/A	N/A	N/A	N/A
**Place of Childbirth (Ref: Home Birth)**							
	Facility Birth	N/A	N/A	N/A	N/A	0.89(0.68–1.11)	0.255
**Parental Variables**							
**Maternal BMI (Ref: Overweight/Obese)**							
	Underweight (<18.50kg/m^2^)	2.01(1.10–3.70)	0.005	2.46(1.55–3.75)	0.000	2.09(1.41–3.09)	<0.001
	Normal (18.50–24.99kg/m^2^)	0.77(0.44–1.37)	0.986	1.53(1.02–2.19)	0.030	1.39(1.05–1.85)	0.021
**Maternal Age (Ref: 35–49 years)**							
	15–24	1.07(0.47–2.43)	0.499	N/A	N/A	0.98(0.52–1.87)	0.958
	25–34	1.11(0.54–2.27)	0.29	N/A	N/A	1.21(0.71–2.08)	0.485
**Maternal Age at First Birth (Ref: <18 years)**							
	19–24	N/A	N/A	N/A	N/A	0.72(0.55–0.94)	0.014
	>25	N/A	N/A	N/A	N/A	0.85(0.47–1.52)	0.575
**Mother’s Education (Ref: Higher)**							
	No Education	10.23(1.54–68.9)	0.008	1.48(0.65–3.34)	0.574	1.98(1.02–3.83)	0.043
	Primary	5.70(0.89–36.50)	0.077	1.15(0.57–2.34)	0.967	1.80(1.15–2.83)	0.010
	Secondary	4.96(0.78–31.50)	0.127	1.46(0.74–2.88)	0.451	1.49(1.03–2.15)	0.036
**Mother’s exposure to Television (Ref: No)**							
	Yes	1.09(0.829–1.44)	0.529	N/A	N/A	N/A	N/A
**Mother’s ANC Visit Number (Ref: ≥4)**							
	Nil	1.26(0.79–2.00)	0.356	0.90(0.54–1.52)	0.502	0.78(0.5–1.23)	0.285
	1–3	0.86(0.58–1.26)	0.351	1.09(0.74–1.59)	0.945	0.96(0.75–1.24)	0.760
**Father’s Education (Ref: Higher)**							
	No Education	2.87(0.93–8.84)	0.284	1.50(0.75–3.02)	0.215	0.94(0.55–1.62)	0.825
	Primary	1.52(0.51–4.58)	0.569	1.56(0.81–2.98)	0.149	1.45(0.98–2.13)	0.060
	Secondary	1.44(0.47–4.45)	0.760	1.29(0.67–2.48)	0.365	1.04(0.74–1.48)	0.817
**Father’s Occupation (Ref: Other)**							
	Agricultural	N/A	N/A	0.94(0.64–1.38)	0.678	0.56(0.34–0.91)	0.019
	Business	N/A	N/A	0.73(0.47–1.23)	0.137	0.61(0.40–0.91)	0.017
	Non-Agricultural	N/A	N/A	0.79(0.54–1.14)	0.211	0.64(0.44–0.93)	0.020

N/A stands for not applicable. Insignificant variables in Chi-square tests were not included in the adjusted model; therefore, they were replaced with N/A in the Table.

p-value <0.05 is the level of significance.

AOR: Adjusted odds ratio; p-value: Probability value; CI: Confidence interval; Ref: Reference category; BMI: Body mass index; ANC: Antenatal care.

Factors associated with wasting within different categories of wealth statuses are reported in **Table 5**. It was evident from the results that only poor households had a higher risk of having wasted children when the mother was underweight. However, mother’s education, type of toilet, ANC visit number, sex, and birth order children had no significant impact on wasting within different wealth statuses. Well-off families residing in Dhaka had a higher chance of having a wasted child, but other household statuses yielded insignificant results. Recent fever experience in the last two weeks was associated with poor and well-off wealth indexes, and it had higher odds of a wasted child.

**Table 5:** Factors associated with wasting within different categories of wealth status.

Independent Variables		Poorest		Poor		Well-Off	
Child-related Variables		AOR (95% CI)	p-value	AOR (95% CI)	p-value	AOR (95% CI)	p-value
**Birth Order (Ref: ≥4)**							
	1^st^	1.45(0.87–2.44)	0.157	N/A	N/A	N/A	N/A
	2^nd^	1.23(0.74–2.05)	0.421	N/A	N/A	N/A	N/A
	3^rd^	1.69(1.00–2.83)	0.049	N/A	N/A	N/A	N/A
**Age (Ref: 0–5 months)**							
	6–11	N/A	N/A	N/A	N/A	1.14(0.82–1.59)	0.424
	12–23	N/A	N/A	N/A	N/A	0.57(0.37–0.88)	0.012
	24–35	N/A	N/A	N/A	N/A	0.65(0.48–0.89)	0.008
	36–59	N/A	N/A	N/A	N/A	0.90(0.67–1.22)	0.489
**Sex (Ref: Male)**							
	Female	1.50(1.08–2.07)	0.015	N/A	N/A	N/A	N/A
**Recent Experience of Fever (Ref: No)**							
	Yes	N/A	N/A	1.44(1.00–2.07)	0.052	1.53(1.22–1.91)	<0.001
**Socioeconomic and Demographic Variables**							
**Division (Ref: Sylhet)**							
	Barisal	N/A	N/A	0.59(0.22–1.59)	0.297	0.76(0.48–1.22)	0.258
	Chittagong	N/A	N/A	0.59(0.28–1.23)	0.161	0.85(0.60–1.22)	0.381
	Dhaka	N/A	N/A	1.09(0.55–2.15)	0.805	0.95(0.67–1.34)	0.780
	Khulna	N/A	N/A	1.46(0.72–2.98)	0.299	0.48(0.30–0.76)	0.002
	Mymensingh	N/A	N/A	1.05(0.52–2.13)	0.897	0.80(0.52–1.25)	0.333
	Rajshahi	N/A	N/A	0.91(0.45–1.85)	0.798	0.59(0.38–0.94)	0.026
	Rangpur	N/A	N/A	0.64(0.30–1.37)	0.255	0.70(0.43–1.15)	0.157
**Place of Residence (Ref: Urban)**							
	Rural	N/A	N/A	1.90(1.23–3.21)	0.016	N/A	N/A
**Type of Toilet (Ref: Hygienic)**							
	Unhygienic	N/A	N/A	1.62(1.14–2.32)	0.008	N/A	N/A
**Parental Variables**							
**Maternal BMI (Ref: Overweight/Obese)**							
	Underweight (<18.50kg/m^2^)	1.47(0.86–2.54)	0.163	2.31(1.21–4.40)	0.011	2.65(1.88–3.75)	0.000
	Normal (18.50–24.99kg/m^2^)	0.84(0.51–1.39)	0.504	1.33(0.74–2.39)	0.343	1.31(1.01–1.69)	0.039
**Mother’s Education (Ref: Higher)**							
	No Education	2.78(0.71–10.86)	0.143	N/A	N/A	1.64(0.96–2.81)	0.072
	Primary	1.67(0.45–6.26)	0.445	N/A	N/A	1.22(0.86–1.73)	0.265
	Secondary	2.01(0.54–7.47)	0.299	N/A	N/A	1.27(0.95–1.69)	0.114
**Mother’s ANC Visit Number (Ref: ≥4)**							
	Nil	N/A	N/A	1.05(0.65–1.69)	0.834	N/A	N/A
	1–3	N/A	N/A	1.14(0.70–1.88)	0.600	N/A	N/A
**Father’s Occupation (Ref: Other)**						
	Agricultural	N/A	N/A	N/A	N/A	1.12(0.72–1.75)	0.610
	Business	N/A	N/A	N/A	N/A	0.62(0.41–0.94)	0.023
	Non-Agricultural	N/A	N/A	N/A	N/A	0.81(0.56–1.17)	0.252

N/A stands for not applicable. Insignificant variables in Chi-square tests were not included in the adjusted model; therefore, they were replaced with N/A in the Table.

p-value <0.05 is the level of significance.

AOR: Adjusted odds ratio; p-value: Probability value; CI: Confidence interval; Ref: Reference category; BMI: Body mass index; ANC: Antenatal care.

**Fig 2:**
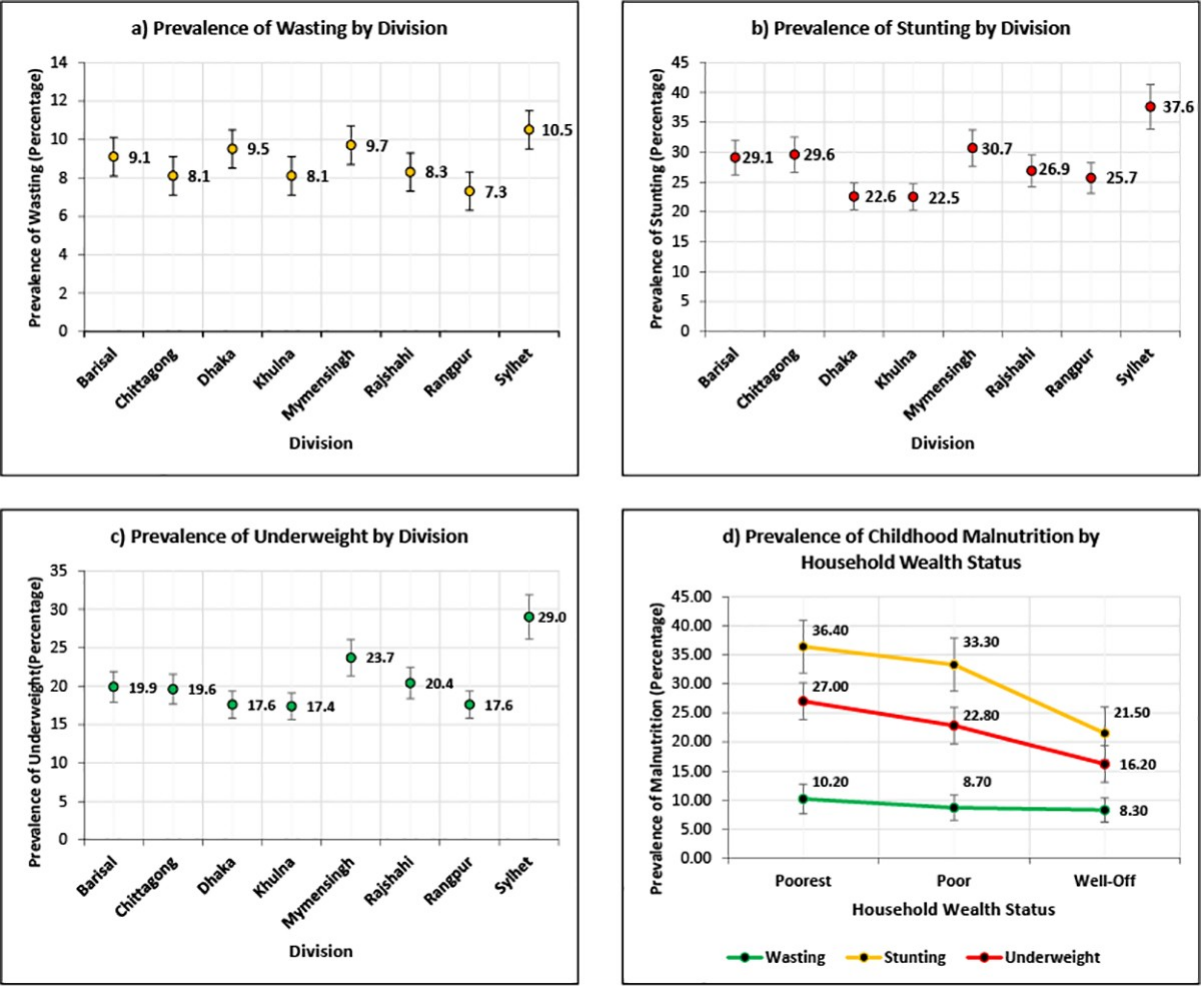
Prevalence of malnutrition by division and household wealth status of Bangladesh.

Fig 2 exhibits the prevalence of different forms of malnutrition by place of residence and different wealth statuses. It is very clear from the figure that stunting, wasting, and underweight prevalence is higher in the poorest, followed by poor and well-off households. The prevalence of these three forms of malnutrition was also comparatively higher in Sylhet division.

## Discussion

The paper aimed to identify the associated factors with childhood malnutrition and poverty in Bangladesh. In low resource countries like Bangladesh, poverty and childhood malnutrition continue to be major public health concerns. Although Bangladesh has attained excellent progress over the past few decades in different areas of health, particularly in health-related Millennium Development Goals, and has made few nutritional achievements [1, 24]. Nevertheless, the high prevalence of childhood malnutrition still exists in Bangladesh, and it cannot be denied [1]. Despite the recent economic growth and poverty reduction, the reduction rate of childhood malnutrition in Bangladesh is somewhat unsatisfactory. There is consensus that economic growth and poverty reduction alone cannot decrease malnutrition [25, 26] unless the issues of mothers’ malnutrition history, teenage marriage, and inappropriate diets are addressed. According to the BDHS 2017–18 report, 31% of under-five children are still stunted and 9% are severely stunted; while 8% are wasted, with 2% being severely wasted and 22% of children are underweight, and 4% are severely underweight [13]. In Bangladesh, 21.8% of population are still living below the national poverty line [27], like many other LMICs, which is predicted to further increase due to the recent COVID-19 pandemic [28]. Besides, the ongoing pandemic is expected to increase the prevalence of childhood malnutrition [29]. Therefore, examining the associated factors with malnutrition and poverty is an enormously important topic in the current context. This is the first study in this context, using the most recent round BDHS 2017–18 dataset to the best of our knowledge.

In terms of childhood malnutrition, a clear rural-urban gap was observed in the current study. Rural children were more likely to be stunted than their urban counterparts (29.2% compared with 21.7%). Stunting was most prevalent in Sylhet (37.6%) and lowest in Dhaka and Khulna. The differences in stunting across wealth quintiles were larger (36.4%) for children whose mothers were in the lowest wealth quintile than their well-off counterparts (21.5%). Children living in rural areas were more likely to be underweighted than those living in urban areas (20.5% vs. 18.4%). A similar negative association was observed between household wealth status and the proportion of underweight children, i.e., children in the poorest households were more likely to be underweight (27.0%) than children from the well-off households (16.2%). The prevalence of wasting was slightly higher among children residing in urban areas (9.7% vs. 8.8%).

The multivariate logistic regression analyses showed several predictor variables were associated with childhood malnutrition across different wealth statuses, which is in line with previous studies in Bangladesh [1–3, 18, 30] and elsewhere [4, 7, 24, 31–33]. The results demonstrate that children from the poorest and poor households were more likely to be stunted, wasted, and underweight. There remains a very close connection between poverty and childhood malnutrition, and vice-versa. Poverty often leads to financial shortages and inadequate basic amenities (such as education, health care services, food insecurity, shelter, etc.), forcing them to live impoverished lives. Worldwide, the poorest countries face the most significant burden of various types of childhood malnutrition [26]. Nutritional disparities reduce productivity and potentially decrease human capital, making countries prone to poverty and reinforcing childhood malnutrition and poverty’s vicious cause-effects cycle [26].

Lack of formal parental education is strongly linked to stunting, wasting, and underweight among under-five children, supporting previous studies conducted in Bangladesh [2, 3, 18, 34] and similar settings [24, 31, 32]. The possible explanation could be that a lower level of education prevents parents from obtaining employment or leads to poorly paid employment, limiting the household incomes and reduces the purchasing capability of good quality and quantity of foods and routine medical check-ups; consequently, children suffer from various infections and parasitic diseases [35]. Furthermore, poor maternal BMI was associated with childhood malnutrition among different categories of wealth statuses, aligning the results with previous studies [1, 3, 18]. As mothers with standard BMI are likely to have healthier babies [36], maternal nutritional status needs to be included in child undernutrition policies and programs. Furthermore, stunting was significantly prevalent among children of advancing age from the poorest and poor families but insignificant for well-off households, supported by a previous study in Nigeria [37]. During the first 11 months, children are being breastfed, and nutrition from the breast milk may help them have adequate growth, but later, when they give up breast milk, the likelihood of childhood stunting increased tremendously [1].

The coexistence of poverty and malnutrition is intergenerational, and this needs to be recognized urgently by implementing effective measures to break this vicious cycle [38–40]. Prior research also showed that malnourished mothers are more likely to have malnourished children [8, 26, 41–43], and early intervention can break this cycle of poverty and childhood malnutrition. The government of Bangladesh is well conscious about this and is doing a lot in this regard. Besides, different NGOs are relentlessly working to ensure nutrition for mothers and children by implementing different programs such as Infant and Young Child Feeding, National Nutrition Programme, Suchana etc. This high prevalence of childhood malnutrition, however, questions the appropriateness of these interventions those were previously implemented.

This study has several policy implications. First, since poverty and malnutrition are intertwined, nutritional and poverty reduction strategies and initiatives should therefore be deserving of preference and should be rounded and holistic in any way. Second, the government should take robust and rigorous steps in improving nutritional status among women and their children to provide good nutritional foods for poor and underprivileged households. Third, interventions must target economic empowerment and short-term dietary supplements for people with disadvantaged economic status. Fourth, the Bangladeshi Government may wish to establish effective policies and programs in collaboration with national and international organizations, which will aid all poor women and infants during pregnancy and postnatal period. Furthermore, parents need better access to health information and education; therefore, community-based health services should be promoted at the grassroots level.

The main strengths of this study are that it used the latest nationwide DHS survey data. The surveys were carried out at the population level with a large study sample, and the results can be generalized for the whole Bangladeshi population. In addition, the appropriate statistical techniques were applied in this study for estimation. However, the limitation of this study is the cross-sectional survey design where both exposure/predictors and outcomes were measured at the same timepoint. Therefore, no causal relationships can be inferred.

## Conclusions

Our investigation has established the significant associated factors with poverty and childhood malnutrition. The study supports the need for organized efforts, especially among the poorest and poor, to reduce the degree of malnutrition. It is well-known that 12 of the 17 SDGs goals prioritize and include highly relevant indicators to nutrition, while the second-highest priority is to end poverty, hunger, food insecurity, and improved nutrition. Therefore, if we want to eradicate poverty and hunger sustainably, we need to build a systemic, healthy, and equitable society.
