# Correction: Poverty and childhood malnutrition: Evidence-based on a nationally representative survey of Bangladesh

**DOI:** 10.1371/journal.pone.0261420

**Published:** 2021-12-09

**Authors:** Md. Ashfikur Rahman, Henry Ratul Halder, Md. Sazedur Rahman, Mahmood Parvez

The Author Contributions for the second author Henry Ratul Halder are incorrect. The publisher apologizes for this error. The contributions of this author are as follows: Conceptualization, Data curation, Formal analysis, Methodology, Software, Writing–original draft, Writing–review & editing.

The affiliation for the third author is incorrect. Md. Sazedur Rahman is not affiliated with #3 but with #2: Statistics Discipline, Science, Engineering and Technology School, Khulna University, Khulna, Bangladesh.

There are errors in Tables 3, 4 and 5. Please see the correct Tables here.

**Table 3:** Factors associated with stunting within different wealth status.

Independent Variables	Poorest		Poor		Well-off	
	AOR (95% CI)	p-value	AOR (95% CI)	p-value	AOR (95% CI)	p-value
**Child-related Variables**						
**Birth Order (Ref: ≥4** ^**th**^ **)**						
1^st^	0.93(0.68–1.29)	0.677	0.65(0.39–1.07)	0.090	0.91(0.63–1.32)	0.615
2^nd^	0.87(0.64–1.19)	0.382	0.58(0.38–0.89)	0.012	0.86(0.60–1.23)	0.405
3^rd^	0.92(0.66–1.29)	0.639	0.68(0.45–1.03)	0.067	0.93(0.63–1.37)	0.700
**Age (months) (Ref: 0–5)**						
6–11	1.08(0.63–1.86)	0.782	0.11(0.05–0.22)	<0.001	0.10(0.01–0.81)	0.031
12–23	2.92(0.63–1.86)	0.892	0.38(0.22–0.67)	0.001	0.14(0.02–1.11)	0.062
24–35	4.41(1.88–4.55)	<0.001	1.23(0.71–1.79)	0.616	0.34(0.04–2.63)	0.300
36–59	3.11(1.90–5.09)	<0.001	1.62(1.06–2.47)	0.026	0.37(0.5–2.91)	0.348
**Socioeconomic and Demographic Variables**						
**Division (Ref: Sylhet)**						
Barisal	0.83(0.53–1.30)	0.413	N/A	N/A	1.10(0.72–1.69)	0.652
Chittagong	1.16(0.79–1.71)	0.445	N/A	N/A	1.04(0.74–1.45)	0.819
Dhaka	0.77(0.51–1.18)	0.229	N/A	N/A	0.79(0.55–1.13)	0.191
Khulna	0.41(0.26–0.71)	0.001	N/A	N/A	1.01(0.67–1.50)	0.981
Mymensingh	0.75(0.50–1.14)	0.184	N/A	N/A	1.01(0.66–1.52)	0.997
Rajshahi	0.58(0.37–0.89)	0.014	N/A	N/A	1.02(0.68–1.54)	0.916
Rangpur	0.52(0.35–0.77)	0.001	N/A	N/A	1.11(0.71–1.75)	0.645
**Place of residence (Ref: Rural)**						
Urban	N/A	N/A	N/A	N/A	0.94(0.76–1.16)	0.543
**Drinking Water Source (Ref: Improved)**						
Unimproved	N/A	N/A	N/A	N/A	1.33(0.54–3.29)	0.540
**Type of Toilet (Ref: Hygienic)**						
Unhygienic	N/A	N/A	1.06(0.84–1.33)	0.624	1.12(0.83–1.50)	0.474
**Place of Childbirth (Ref: Home Delivery)**						
Facility Birth	N/A	N/A	N/A	N/A	0.89(0.72–1.11)	0.307
**Parental Variables**						
**Maternal BMI (Ref: Overweight/Obese)**						
Underweight (<18.50kg/m^2^)	1.60(1.09–2.35)	0.016	2.11(1.43–2.23)	<0.001	1.35(0.96–1.90)	0.083
Normal (18.50–24.99kg/m^2^)	1.14(0.81–1.59)	0.458	1.70(1.21–2.39)	0.002	1.02(0.81–1.29)	0.850
**Maternal Age at First Birth (Ref: <18 years)**						
19–24	N/A	N/A	N/A	N/A	0.90(0.73–1.11)	0.332
≥25	N/A	N/A	N/A	N/A	0.91(0.58–1.42)	0.662
**Mother’s Education (Ref: Higher)**						
No Education	1.99(0.83–4.80)	0.123	2.41(1.22–6.58)	0.016	1.70(0.96–3.02)	0.071
Primary	1.60(0.70–3.66)	0.269	3.72(1.78–7.67)	<0.001	1.55(1.05–2.72)	0.027
Secondary	1.66(0.73–3.78)	0.226	3.51(1.70–6.94)	0.001	1.41(1.03–1.91)	0.030
**Mother’s Current Working Status (Ref: Yes)**						
No	N/A	N/A	N/A	N/A	0.88(0.70–1.90)	0.241
**Mother’s Exposure to Television (Ref: No)**						
Yes	N/A	N/A	N/A	N/A	0.99(0.78–1.27)	0.985
**Mother’s ANC Visit Number (Ref: ≥4)**						
Nil	0.76(0.51–1.14)	0.296	1.16(0.74–1.84)	0.514	1.22(0.84–1.77)	0.295
1–3	0.70(0.51–0.98)	0.028	1.23(0.89–1.72)	0.214	1.20(0.97–1.49)	0.093
**Father’s Education Level (Ref: Higher)**						
No Education	2.94(1.37–6.34)	0.006	1.45(0.81–2.60)	0.216	1.98(1.26–3.11)	0.003
Primary	2.41(1.15–5.10)	0.021	1.01(0.59–1.74)	0.960	1.89(1.34–2.67)	<0.001
Secondary	2.18(1.02–4.67)	0.045	0.87(0.50–1.50)	0.614	1.51(1.12–2.04)	0.008
**Father’s Occupation (Ref: Other)**						
Agricultural	N/A	N/A	N/A	N/A	0.82(0.54–1.25)	0.351
Business	N/A	N/A	N/A	N/A	0.70(0.48–1.01)	0.055
Non-Agricultural	N/A	N/A	N/A	N/A	0.71(0.51–1.00)	0.050

N/A stands for not applicable. Insignificant variables in Chi-square tests were not included in the adjusted model; therefore, they were replaced with N/A in the Table.

p-value <0.05 is the level of significance.

AOR: Adjusted odds ratio; p-value: Probability value; CI: Confidence interval; Ref: Reference category; BMI: Body mass index; ANC: Antenatal care.

**Table 4:** Factors associated with underweight within different categories of wealth status.

	Poorest		Poor		Well-Off	
Independent Variables	AOR (95% CI)	p-value	AOR (95% CI)	p-value	AOR (95% CI)	p-value
**Child-related Variables**						
**Birth Order (Ref: ≥4** ^**th**^ **)**						
1^st^	1.55(0.83–2.90)	0.864	N/A	N/A	0.88(0.51–1.52)	0.634
2^nd^	1.04(0.60–1.80)	0.621	N/A	N/A	0.74(0.47–1.17)	0.197
3^rd^	0.97(0.57–1.63)	0.552	N/A	N/A	0.71(0.45–1.12)	0.137
**Age (Ref: 0–5 months)**						
6–11	1.68(0.90–3.16)	0.105	2.60(1.27–5.34)	0.001	0.24(0.02–2.51)	0.231
12–23	2.95(1.71–5.08)	<0.001	3.11(1.61–6.00)	0.001	0.21(0.02–2.30)	0.204
24–35	3.50(2.05–6.00)	<0.001	5.72(0.98–6.01)	0.193	0.29(0.03–3.05)	0.302
36–59	1.68(0.90–3.16)	0.290	7.37(3.49–15.55)	0.001	0.51(0.05–5.29)	0.568
**Recent Experience of Fever (Ref: No)**						
Yes	N/A	N/A	1.51(1.16–1.95)	0.002	1.30(1.03–1.64)	0.027
**Socioeconomic and Demographic Variables**						
**Division (Ref: Sylhet)**						
Barisal	1.07(0.56–2.03)	0.995	0.53(0.28–0.99)	0.040	0.55(0.32–0.94)	0.030
Chittagong	0.91(0.52–1.59)	0.771	0.55(0.34–0.89)	0.008	0.89(0.61–1.30)	0.538
Dhaka	1.06(0.56–2.02)	0.523	0.62(0.38–1.01)	0.056	0.68(0.46–1.01)	0.058
Khulna	0.82(0.37–1.82)	0.242	0.67(0.39–1.15)	0.122	0.78(0.50–1.22)	0.278
Mymensingh	0.85(0.46–1.57)	0.864	0.85(0.52–1.40)	0.367	0.84(0.53–1.32)	0.444
Rajshahi	0.60(0.30–1.21)	0.412	0.58(0.35–0.96)	0.037	0.74(0.46–1.17)	0.196
Rangpur	0.79(0.45–1.41)	0.440	0.42(0.25–0.71)	0.001	0.86(0.52–1.43)	0.554
**Place of Residence (Ref: Urban)**						
Rural	N/A	N/A	0.71(0.47–1.08)	0.079	N/A	N/A
**Type of Toilet (Ref: Hygienic)**						
Unhygienic	1.25(0.90–1.74)	0.301	N/A	N/A	N/A	N/A
**Place of Childbirth (Ref: Home Birth)**						
Facility Birth	N/A	N/A	N/A	N/A	0.89(0.68–1.11)	0.255
**Parental Variables**						
**Maternal BMI (Ref: Overweight/Obese)**						
Underweight (<18.50kg/m^2^)	2.01(1.10–3.70)	0.005	2.46(1.55–3.75)	<0.001	2.09(1.41–3.09)	<0.001
Normal (18.50–24.99kg/m^2^)	0.77(0.44–1.37)	0.986	1.53(1.02–2.19)	0.030	1.39(1.05–1.85)	0.021
**Maternal Age (Ref: 35–49 years)**						
15–24	1.07(0.47–2.43)	0.499	N/A	N/A	0.98(0.52–1.87)	0.958
25–34	1.11(0.54–2.27)	0.29	N/A	N/A	1.21(0.71–2.08)	0.485
**Maternal Age at First Birth (Ref: <18 years)**						
19–24	N/A	N/A	N/A	N/A	0.72(0.55–0.94)	0.014
>25	N/A	N/A	N/A	N/A	0.85(0.47–1.52)	0.575
**Mother’s Education (Ref: Higher)**						
No Education	10.23(1.54–68.9)	0.008	1.48(0.65–3.34)	0.574	1.98(1.02–3.83)	0.043
Primary	5.70(0.89–36.50)	0.077	1.15(0.57–2.34)	0.967	1.80(1.15–2.83)	0.010
Secondary	4.96(0.78–31.50)	0.127	1.46(0.74–2.88)	0.451	1.49(1.03–2.15)	0.036
**Mother’s exposure to Television (Ref: No)**						
Yes	1.09(0.829–1.44)	0.529	N/A	N/A	N/A	N/A
**Mother’s ANC Visit Number (Ref: ≥4)**						
Nil	1.26(0.79–2.00)	0.356	0.90(0.54–1.52)	0.502	0.78(0.5–1.23)	0.285
1–3	0.86(0.58–1.26)	0.351	1.09(0.74–1.59)	0.945	0.96(0.75–1.24)	0.760
**Father’s Education (Ref: Higher)**						
No Education	2.87(0.93–8.84)	0.284	1.50(0.75–3.02)	0.215	0.94(0.55–1.62)	0.825
Primary	1.52(0.51–4.58)	0.569	1.56(0.81–2.98)	0.149	1.45(0.98–2.13)	0.060
Secondary	1.44(0.47–4.45)	0.760	1.29(0.67–2.48)	0.365	1.04(0.74–1.48)	0.817
**Father’s Occupation (Ref: Other)**						
Agricultural	N/A	N/A	0.94(0.64–1.38)	0.678	0.56(0.34–0.91)	0.019
Business	N/A	N/A	0.73(0.47–1.23)	0.137	0.61(0.40–0.91)	0.017
Non-Agricultural	N/A	N/A	0.79(0.54–1.14)	0.211	0.64(0.44–0.93)	0.020

N/A stands for not applicable. Insignificant variables in Chi-square tests were not included in the adjusted model; therefore, they were replaced with N/A in the Table.

p-value <0.05 is the level of significance.

AOR: Adjusted odds ratio; p-value: Probability value; CI: Confidence interval; Ref: Reference category; BMI: Body mass index; ANC: Antenatal care.

**Table 5:** Factors associated with wasting within different categories of wealth status.

Independent Variables	Poorest		Poor		Well-Off	
Child-related Variables	AOR (95% CI)	p-value	AOR (95% CI)	p-value	AOR (95% CI)	p-value
**Birth Order (Ref: ≥4** ^**th**^ **)**						
1^st^	1.45(0.87–2.44)	0.157	N/A	N/A	N/A	N/A
2^nd^	1.23(0.74–2.05)	0.421	N/A	N/A	N/A	N/A
3^rd^	1.69(1.00–2.83)	0.050	N/A	N/A	N/A	N/A
**Age (Ref: 0–5 months)**						
6–11	N/A	N/A	N/A	N/A	1.14(0.82–1.59)	0.424
12–23	N/A	N/A	N/A	N/A	0.57(0.37–0.88)	0.012
24–35	N/A	N/A	N/A	N/A	0.65(0.48–0.89)	0.008
36–59	N/A	N/A	N/A	N/A	0.90(0.67–1.22)	0.489
**Sex (Ref: Male)**						
Female	1.50(1.08–2.07)	0.015	N/A	N/A	N/A	N/A
**Recent Experience of Fever (Ref: No)**						
Yes	N/A	N/A	1.44(1.00–2.07)	0.052	1.53(1.22–1.91)	<0.001
**Socioeconomic and Demographic Variables**						
**Division (Ref: Sylhet)**						
Barisal	N/A	N/A	0.59(0.22–1.59)	0.297	0.76(0.48–1.22)	0.258
Chittagong	N/A	N/A	0.59(0.28–1.23)	0.161	0.85(0.60–1.22)	0.381
Dhaka	N/A	N/A	1.09(0.55–2.15)	0.805	0.95(0.67–1.34)	0.780
Khulna	N/A	N/A	1.46(0.72–2.98)	0.299	0.48(0.30–0.76)	0.002
Mymensingh	N/A	N/A	1.05(0.52–2.13)	0.897	0.80(0.52–1.25)	0.333
Rajshahi	N/A	N/A	0.91(0.45–1.85)	0.798	0.59(0.38–0.94)	0.026
Rangpur	N/A	N/A	0.64(0.30–1.37)	0.255	0.70(0.43–1.15)	0.157
**Place of Residence (Ref: Urban)**						
Rural	N/A	N/A	1.90(1.23–3.21)	0.016	N/A	N/A
**Type of Toilet (Ref: Hygienic)**						
Unhygienic	N/A	N/A	1.62(1.14–2.32)	0.008	N/A	N/A
**Parental Variables**						
**Maternal BMI (Ref: Overweight/Obese)**						
Underweight (<18.50kg/m^2^)	1.47(0.86–2.54)	0.163	2.31(1.21–4.40)	0.011	2.65(1.88–3.75)	<0.001
Normal (18.50–24.99kg/m^2^)	0.84(0.51–1.39)	0.504	1.33(0.74–2.39)	0.343	1.31(1.01–1.69)	0.059
**Mother’s Education (Ref: Higher)**						
No Education	2.78(0.71–10.86)	0.143	N/A	N/A	1.64(0.96–2.81)	0.720
Primary	1.67(0.45–6.26)	0.445	N/A	N/A	1.22(0.86–1.73)	0.265
Secondary	2.01(0.54–7.47)	0.299	N/A	N/A	1.27(0.95–1.69)	0.114
**Mother’s ANC Visit Number (Ref: ≥4)**						
Nil	N/A	N/A	1.05(0.65–1.69)	0.834	N/A	N/A
1–3	N/A	N/A	1.14(0.70–1.88)	0.600	N/A	N/A
**Father’s Occupation (Ref: Other)**						
Agricultural	N/A	N/A	N/A	N/A	1.12(0.72–1.75)	0.610
Business	N/A	N/A	N/A	N/A	0.62(0.41–0.94)	0.023
Non-Agricultural	N/A	N/A	N/A	N/A	0.81(0.56–1.17)	0.252

N/A stands for not applicable. Insignificant variables in Chi-square tests were not included in the adjusted model; therefore, they were replaced with N/A in the Table.

p-value <0.05 is the level of significance.

AOR: Adjusted odds ratio; p-value: Probability value; CI: Confidence interval; Ref: Reference category; BMI: Body mass index; ANC: Antenatal care.
